# Development of clinical prediction model to guide the use of CT head scans for non-traumatic Thai patient with seizure: A cross-sectional study

**DOI:** 10.1371/journal.pone.0305484

**Published:** 2024-07-10

**Authors:** Pimploy Suriyanusorn, Thanin Lokeskrawee, Jayanton Patumanond, Suppachai Lawanaskol, Pakpoom Wongyikul

**Affiliations:** 1 Department of Emergency Medicine, Lampang Hospital, Muang District, Lampang, Thailand; 2 Center for Clinical Epidemiology and Clinical Statistics, Faculty of Medicine, Chiang Mai University, Chiang Mai, Thailand; 3 Chaiprakarn Hospital, Chiang Mai, Thailand; Rutland Regional Medical Center, UNITED STATES

## Abstract

The aim of this study was to develop clinical predictor tools for guiding the use of computed tomography (CT) head scans in non-traumatic Thai patients presented with seizure. A prediction model using a retrospective cross-sectional design was conducted. We recruited adult patients (aged ≥ 18 years) who had been diagnosed with seizures by their physicians and had undergone CT head scans for further investigation. Positive CT head defined as the presence of any new lesion that related to the patient’s presented seizure officially reported by radiologist. A total of 9 candidate predictors were preselected. The prediction model was developed using a full multivariable logistic regression with backward stepwise elimination. We evaluated the model’s predictive performance in terms of its discriminative ability and calibration via AuROC and calibration plot. The application was then constructed based on final model. A total of 362 patients were included into the analysis which comprising of 71 patients with positive CT head findings and 291 patients with normal results. Six final predictors were identified including: Glasgow coma scale, the presence of focal neurological deficit, history of malignancy, history of CVA, Epilepsy, and the presence of alcohol withdrawal symptom. In terms of discriminative ability, the final model demonstrated excellent performance (AuROC of 0.82 (95% CI: 0.76–0.87)). The calibration plot illustrated a good agreement between observed and predicted risks. This prediction model offers a reliable tool for effectively reduce unnecessary use and instill confidence in supporting physicians in determining the need for CT head scans in non-traumatic patients with seizures.

## Introduction

Patients experiencing seizures often present to the emergency department (ED), accounting for approximately one-third of neurological conditions and 1% of all ED visits [1, 2]. Seizures refer to the signs and/or symptoms that occur as a consequence of abnormal neuronal activity, which can be caused by various sources, including stroke, tumors, alcohol withdrawal, and infections. According to guidelines [3, 4], the use of a Computed Tomography (CT) head scans as a diagnostic test should be reserved for patient with high risk for positive finding on CT such as old age, with focal neurological deficit, with history of malignancy. However, in routine clinical practice, the decision to perform a CT head scans is left to the discretion of the physician.

Due to increased availability and fast scanning speeds, almost all patients with seizures or a history of symptom related seizures (uncontrolled body stiffening, limb jerking, or sensory aura) usually undergo a CT head scans as part of their workup [5–7]. Based on previous evidence, CT head scans have shown a low yield, ranging from 3.2% to 21.3% [8]. The proportion of management changes after investigation was even lower [8]. Requesting unnecessary CT head scans can lead to excessive costs, burdens on healthcare workers and unnecessary radiation exposure for patient. Additionally, CT head scans are one of the leading causes of radiation exposure, and their association with cancer development is well-documented [9]. The benefits of CT must be carefully considered in relation to the risks posed to both the patient and the healthcare system.

According to surveys [10], decision support tools show promise in helping emergency physicians effectively utilize CT head scans. Many studies have demonstrated the potential factors that associated with positive finding on CT head scans such as Age, focal neurological deficit, history of malignancy, alcohol withdrawal symptoms (AWS) [5, 11–13]. However, studies focusing on the utilization of these factors to develop clinical prediction tools for supporting the use of CT head scans in patients with seizures in resource-constrained settings are limited. Therefore, to assist physician decision-making, the aim of this study was to develop clinical predictor tools for guiding CT head scans in non-traumatic Thai patients presented with seizure.

## Method

### Study design

This study was a diagnostic prediction research with a retrospective cross-sectional design at the Emergency Department of Lampang Hospital, a tertiary referral hospital in Thailand, during November 2019 to September 2021. The study received approval from the institutional review board and ethics committee of Lampang Hospital (No.133/65). Informed consent was waived due to the observational nature of the study. All tests and treatments were ordered as part of standard clinical or laboratory practice, and all were done in the past. Data were collected from December 2021 to March 2022. Patient identity was access only during the data collection and was not collected. This study was reported according to Transparent reporting of a multivariable prediction model for individual prognosis or diagnosis (TRIPOD) reporting guideline [14].

### Participant and data collection

Adult patients (≥ 18 years) who was diagnosed with seizures by their physician and underwent CT head scans for investigation were recruited into the study. Seizures refer to the uncontrolled, abnormal electrical activity of the brain that may cause changes in the level of consciousness, behavior, memory, or feelings [15]. Demographic data, clinical characteristics, CT head scans official reports, and time parameters were retrospectively retrieved from the standardized electronic medical records. Patients without official reports from radiologists and those whose seizures were related to trauma were excluded.

### Candidate predictor

A total of 9 potential predictors were preselected based on previously reported literature, expert consensus, simplicity, and their availability in general ED [5, 11–13, 16]. Demographic data: age, gender, clinical data: Glasgow coma scale (GCS) of 8≤, 9–13, >13, new-onset focal neurological deficit (absence or presence) defined as: a set of symptoms or sign in which causation can be localized to an anatomic site in the central nervous system [17], previous CT head scans status (absence, normal or abnormal), history of malignancy (absence or presence), history of cerebrovascular accident (CVA) (absence or presence), Epilepsy (absence or presence) defined as: two unprovoked seizures occurring more than 24 h apart; a single unprovoked seizure if recurrence risk is high (ie, >60% over the next 10 years); or a diagnosis of an epilepsy syndrome [18], AWS (present or absence) defined as the present of autonomic hyperactivity (sweating, tachycardia); increased hand tremor; insomnia; nausea or vomiting; transient visual, tactile, auditory hallucinations or illusions; psychomotor agitation; anxiety; or tonic-clonic seizures caused by abruptly discontinued of previous large consumed alcohol [19, 20]. All predictors can be accessed via hospital network database at the time of prediction.

### Reference standard

Positive computed tomography (CT) head defined as the presence of any new lesion that related to the patient’s presented seizure officially reported by radiologist.

### Statistical analysis and sample size estimation

All statistical analyses were performed using Stata 17 (StataCorp, Lakeway, Texas, USA). Categorical variables were described using frequency and percentages. Numerical data were assessed for distribution using histograms, and described using means and standard deviations (SD) or medians and interquartile ranges (IQR) based on their distributions. Fisher’s exact test was used for comparison of categorical variables, t-test and Mann Whitney U test were used for continuous variables comparison as appropriate. Statistical test results were considered significant if the p-values were less than 0.05. The sample size for developing a multivariable model with a binary outcome was calculated using the formula proposed by Richard D. Riley [21]. We set the acceptable C-statistic and shrinkage factors at 0.8 and 0.9, respectively, considering 8 potential predictors. The incidence of positive CT head findings from pilot data at Lampang Hospital was 0.2. A minimum sample size of 352 patients and a minimum of 71 patients with positive CT head findings were required.

### Model development

The final prediction model was developed using full multivariable logistic regression with backward stepwise elimination. Initially, all preselected predictors were included, irrespective of their statistical significance in the univariable analysis. Variables that did not show significance at a level of 0.05 were subsequently excluded, starting with the predictor that had the highest P value [22]. All coefficients obtained from the final model were utilized in the generation of the prediction application.

### Model performance and internal validation

The model’s predictive performance was evaluated in terms of discriminative ability and calibration. Discriminative ability was assessed using the C-statistic and presented as the area under the receiver operating characteristic curve (AuROC). According to Hosmer and Lemeshow, an AuROC of 0.70–0.80, 0.80–0.90, and above 0.90 were considered acceptable, excellent, and outstanding, respectively [23]. For calibration, a calibration plot was used to visually inspect the agreement between observed risk and predictions. Internal validation was conducted through bootstrap resampling with 200 replicates to assess model optimism.

To evaluate the clinical utility of the prediction model, we performed decision curve analysis (DCA) [24, 25]. This straightforward method examines the net benefit (NB) derived from employing prediction models to guide clinical decisions. NB is computed by subtracting harms (false positives) from benefits (true positives). DCA were illustrated how the NB of the prediction models varied across different threshold probabilities for patients with positive findings on CT head scans. Considering previous research findings, we selected a range of 2% to 30% as reasonable threshold values.

### Identifying cut point for clinical implication

The diagnostic indices, including sensitivity, specificity, positive predictive value (PPV), negative predictive value (NPV), were calculated for each cut point based on estimated probability. Our aim is to establish cut-point values that can effectively assist clinicians in making decisions for performing CT head scans to achieve a definitive diagnosis, while also focusing on balance between the potential drawbacks of overutilization and the benefits of CT head scans.

## Result

### Study patient

**Fig 1:**
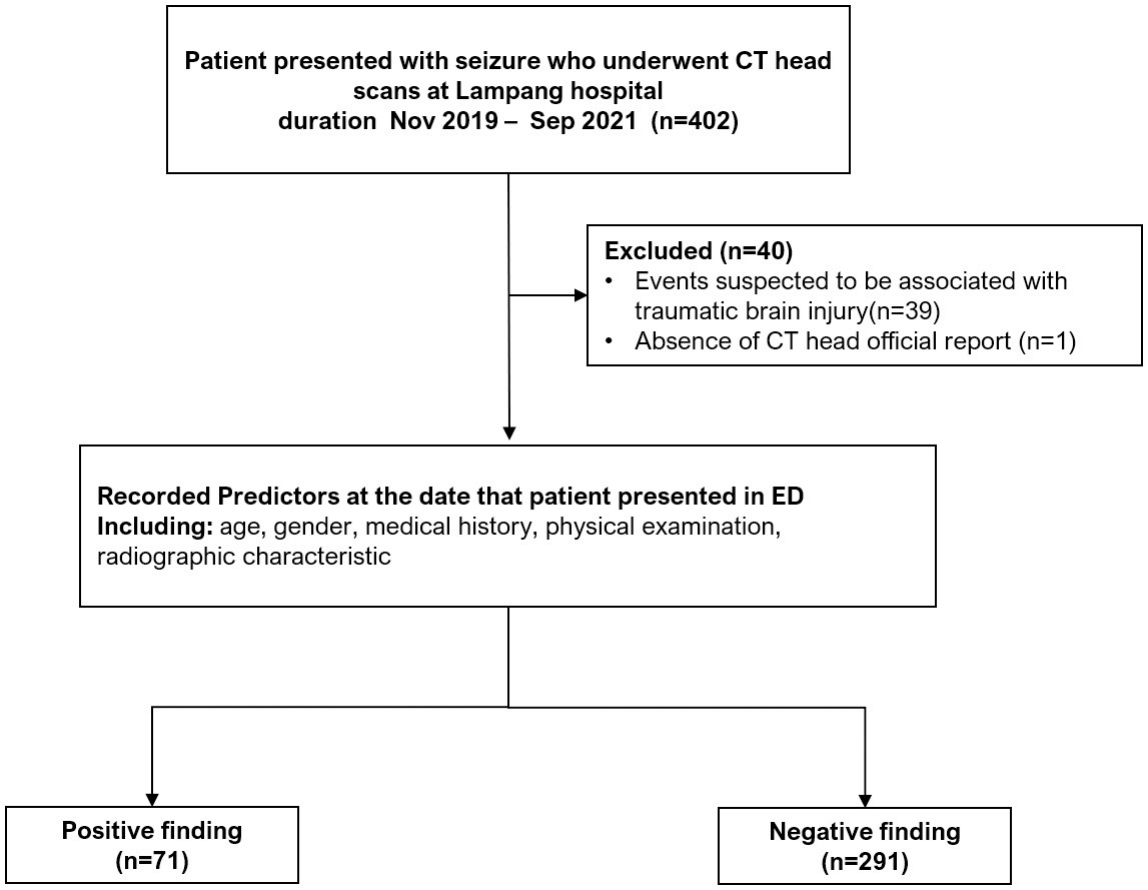
Study flow diagram.

During November 2019 to September 2021, a total of 402 patients presented with seizure. Among these patients, 40 were excluded due to events suspected to be associated with traumatic brain injury (n = 39), and CT head scans were not performed. The remaining 362 patients were included into the analysis which comprising of 71 patients with positive CT head findings and 291 patients with normal results (Fig 1). The overall mean age of the study population was 55.2 ± 16.2 years, with three-quarters being male (73.8%). The prevalence of patients with focal neurological deficits was 17.1%. Approximately half of the total population previously had of CT head scans prior to the seizure event (47.5%).

Table 1 compares baseline characteristic between patient with positive CT head findings and those with normal findings. Statistically significant differences were observed in focal neurological deficits, previous CT head status, history of malignancy, history of CVA, epilepsy, and alcohol withdrawal symptoms. Patients with positive CT head findings exhibited a lower level of GCS compared to those with normal results (GCS median (IQR) 10 (7,15) vs. 15 (11,15)). Additional details of the predictors are presented in Table 1.

**Table 1:** Comparison of baseline characteristics of between patients with positive finding and negative finding.

Characteristics	Total (N = 362) n (%)	Positive N = 71	Negative N = 291	P-value
**Demographic data**				
Male	267 (73.8)	47 (66.2)	220 (75.6)	0.132
Age (year), Mean±SD	55.2±16.2	58.1±14.5	54.5±16.5	0.091
**Clinical finding**				
SBP (mmHg), Mean±SD	141.2±29.2	145.5±35.1	140.2±27.6	0.171
DBP (mmHg), Mean±SD	83.9± 18.6	87.0±22.7	83.2±17.4	0.115
Glasglow Coma Scale				
• Eye, Median [IQR]	4 [3, 4]	4 [2, 4]	4 [4, 4]	<0.001
• Verbal Median [IQR]	5 [1, 5]	2 [1, 5]	5 [2, 5]	<0.001
• Motor Median [IQR]	6 [5, 6]	5 [4, 6]	6 [5, 6]	<0.001
• Total	15 [10, 15]	10 [7, 15]	15 [11, 15]	<0.001
Pupil (mm)	3 [3, 3]	3 [2, 3]	3 [3, 3]	0.978
Focal neurologic deficit	62 (17.1)	27 (38.0)	35 (12.0)	<0.001
**Radiographic data**				
Previous CT				
• No previous CT	172 (47.5)	41 (57.8)	131 (42.0)	0.039
• Normal finding	91 (25.1)	10 (14.1)	81 (27.8)	
• Abnormal finding	99 (27.4)	20 (28.2)	79 (27.2)	
**Medical history**				
History of malignancy	15 (4.1)	10 (14.1)	5 (1.7)	<0.001
Old cerebrovascular accident	74 (20.4)	8 (11.3)	66 (22.7)	0.020
Hypertension	124 (34.3)	23 (32.4)	101 (34.7)	0.413
Diabetes mellitus	65 (17.9)	10 (14.1)	55 (18.9)	0.222
Epilepsy	86 (23.8)	6 (8.5)	80 (27.5)	<0.001
End-stage renal disease	25 (6.9)	4 (5.6)	21 (7.2)	0.435
Using anticoagulant	11 (3.0)	2 (2.8)	9 (3.1)	0.630
Chronic liver disease	5 (1.4)	0 (0.0)	5 (1.7)	0.333
Substance abuse	6 (1.7)	0 (0.0)	6 (2.1)	0.264
Smoking	91 (25.6)	19 (27.5)	72 (25.2)	0.396
Alcohol drinking	148 (41.6)	31 (44.9)	117 (40.8)	0.310
Alcoholic withdrawal symptoms	58 (16.0)	2 (2.9)	56 (19.5)	<0.001

**Abbreviation**: CT, computed tomography; IQR, interquartile range; SD, standard deviation

### Predictor outcome association

Regrading to the result, six predictors showed significant associations with positive CT head findings in the multivariable analysis. GCS of 9–13 and ≤ 8 were associated with odds ratios (OR) of 2.95 (95% confidence interval [CI]: 1.40–6.23) and 5.17 (95% CI: 2.35–11.36), respectively, indicating an increased risk of positive CT head findings. The presence of focal neurological deficits (OR: 2.44, 95% CI: 1.21–4.93) and a history of malignancy (OR: 8.97, 95% CI: 2.34–34.40) were also associated with an increased risk of positive CT head findings (Table 2). Conversely, three predictors, including a history of CVA (OR: 0.36, 95% CI: 0.13–0.99), epilepsy (OR: 0.31, 95% CI: 0.12–0.84), and the presence of alcohol withdrawal symptoms (OR: 0.13, 95% CI: 0.03–0.56), were inversely associated with the likelihood of positive CT head findings (Table 2).

**Table 2:** Multivariable logistic regression analysis of full and final model for diagnostic prediction of positive finding on CT head.

Predictor	Full model		Final model	
	aOR (95% CI)	P-value	aOR (95% CI)	P-value
Male	0.98 (0.50–1.93)	0.957	not included	
Age	0.99 (0.97–1.01)		not included	
Glasgow coma scale				
• GCS >13	reference		reference	
• GCS 9–13	2.95 (1.40–6.23)	<0.001	2.67 (1.30–5.47)	0.007
• GCS ≤8	5.17 (2.35–11.36)	0.024	4.72 (2.19–10.12)	<0.001
Focal neurological deficit	2.44 (1.21–4.93)	0.012	2.41 (1.23–4.72)	0.010
Previous CT status			not included	
• Normal finding	reference			
• Absence previous CT	1.81 (0.75–4.40)	0.189		
• Abnormal finding	1.42 (0.55–3.69)	0.472		
History of malignancy	8.97 (2.34–34.40)	<0.001	8.05 (2.21–29.29)	0.002
History of CVA	0.36 (0.13–0.99)	0.047	0.28 (0.11–0.67)	0.004
Epilepsy	0.31 (0.12–0.84)	0.021	0.29 (0.11–0.72)	0.008
Alcoholic withdrawal symptoms	0.13 (0.03–0.56)	0.006	0.14 (0.03–0.61)	0.009

**Abbreviation**: aOR, adjusted odds ratio; CVA, cerebrovascular accident; GCS, Glasgow coma scale; 95%CI, 95% confident interval

### Model performance

**Fig 2:**
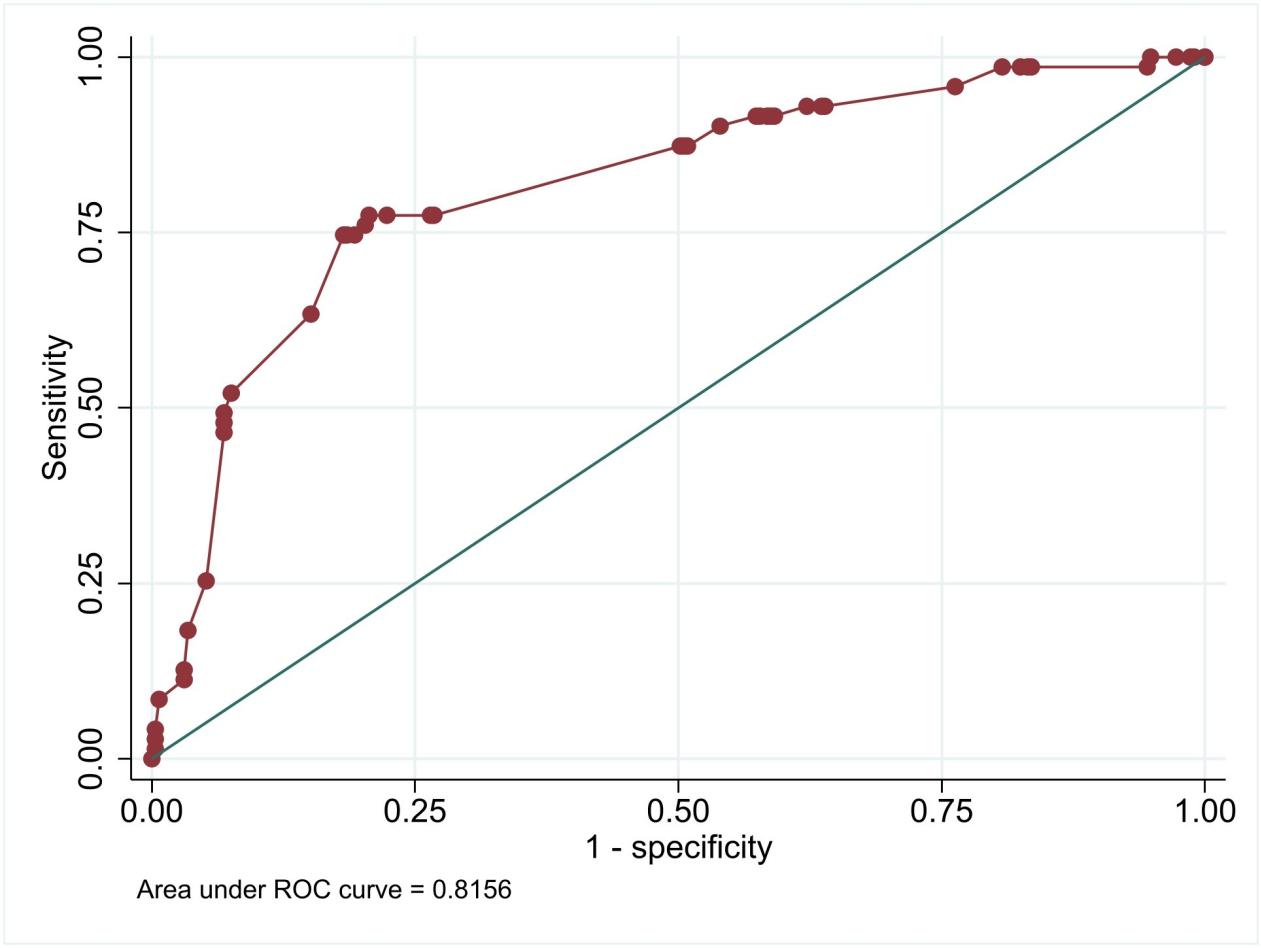
Area under the receiver operating characteristic of final model.

After backward stepwise elimination, 6 final predictors were identified including: GCS, the presence of focal neurological deficit, history of malignancy, history of CVA, Epilepsy, and the presence of AWS. In terms of discriminative ability, the final model demonstrated excellent performance with AuROC of 0.82 (95% CI: 0.76–0.87) (Fig 2). Regarding model calibration, the calibration plot illustrated a good agreement between observed and predicted risks (S1 Fig in S1 File). The C-statistics from bootstrap resampling were 0.80 (95% CI: 0.75–0.86), and the estimated optimism was 0.02 (95% CI: 0.01–0.03). The equation used for probability estimation in developing the application is provided below.

$$\begin{array}{l} predicted\;probability\\ =\;\frac{e^{\left(-1.77+2.09*x1+0.88*x2+0.98*x3+1.55*x4-1.94*x5-1.29*x6-1.25*x7\right)}}{1+e^{\left(-1.77+2.09*x1+0.88*x2+0.98*x3+1.55*x4-1.94*x5-1.29*x6-1.25*x7\right)}} \end{array}$$

X1 denoted a history of malignancy, X2 denoted the presence of focal neurological deficit, X3 denoted GCS (9–13), X4 denoted GCS ≤8, X5 demoted presence of AWS, X6 denoted the history of CVA, X7 denoted epilepsy.

**Fig 3:**
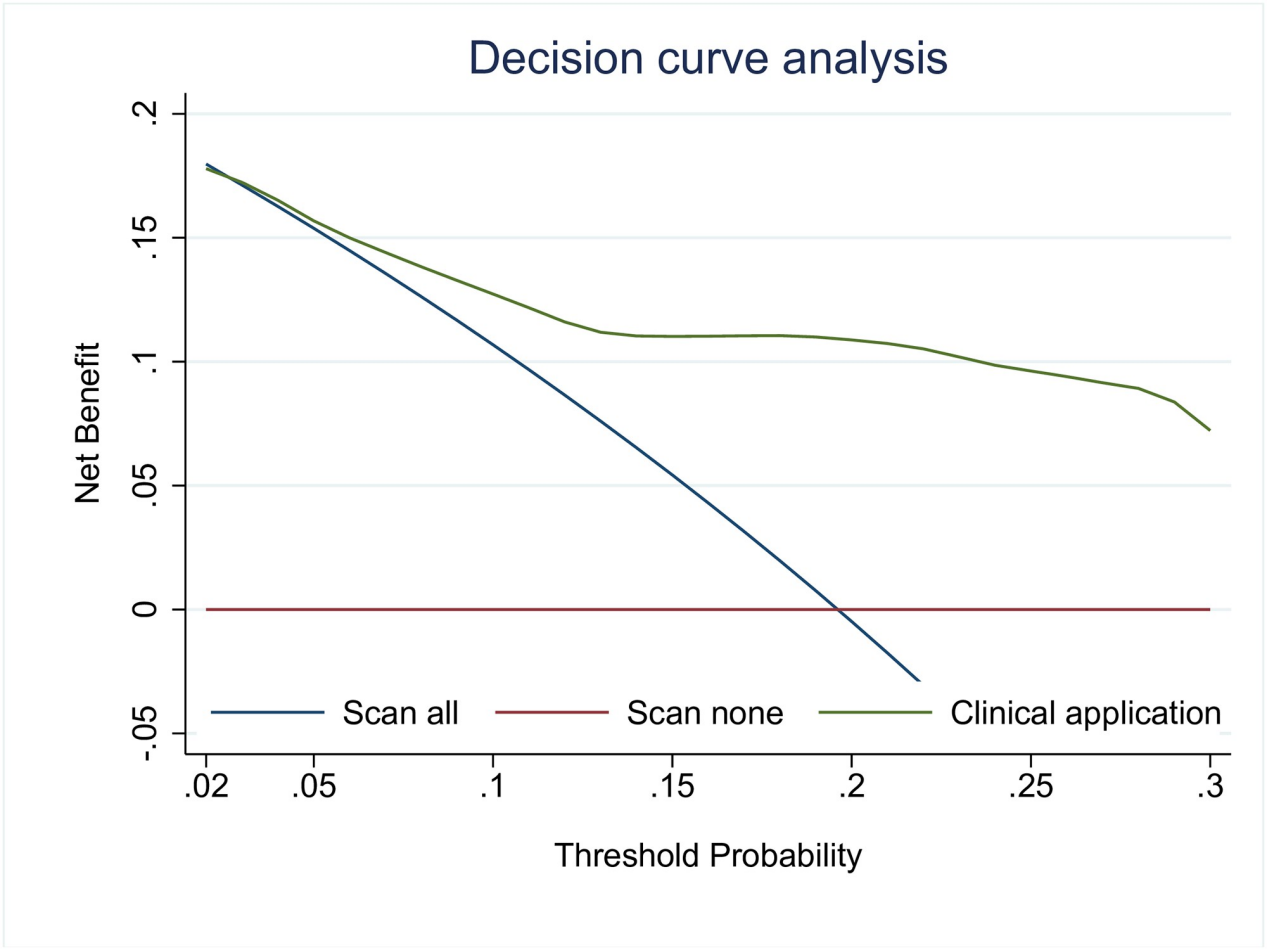
Decision curve illustrating the net benefit of the newly derived clinical prediction model.

The clinical utility of prediction model was illustrated via DCA (Fig 3). The NB of newly derived clinical prediction model was higher than scan all strategies across the entire range of threshold probability for patient with positive finding on CT head scans.

### Cut point threshold selection

Table 3 presents the detailed diagnostic indices at four potential threshold cut-points (15%, 20%, 25%, and 30%). At a probability threshold of 20%, the values of NPV and sensitivity were the highest, at 93.5% and 77.5%, respectively, along with a specificity of 79.4%. Conversely, at a 25% probability threshold, specificity was the highest, at 84.9%, with a sensitivity of 63.4% and NPV of 90.5%. Further details regarding the proportion of observed events and other diagnostic indices can be found in Table 3.

**Table 3:** Number of classified case and diagnostic indices for each cut point based on estimated probability of having positive finding on CT head scans.

Cut point	CT +ve	CT -ve	Total	Sensitivity	Specificity	PPV	NPV
Probability > 15	55	78	133	77.46	73.20	41.35	93.01
Probability ≤ 15	16	213	213				
Probability > 20	55	60	115	77.50	79.40	47.80	93.50
Probability ≤ 20	16	231	247				
Probability > 25	53	54	107	63.40	84.90	50.60	90.50
Probability ≤ 25	18	237	255				
Probability > 30	45	44	89	63.38	84.88	50.56	90.48
Probability ≤ 30	26	247	273				

Abbreviation: CT +ve, true positive; CT -ve, true negative; PPV, positive predictive value; NPV, negative predictive value

## Discussion

In this study, we have developed a clinical prediction model to assist in guiding the utilizing of CT head scans in Thai patients presenting with seizures. The final model incorporates six predictors, including: GCS, the presence of neurological deficits, a history of malignancy, a history of CVA, epilepsy, and the presence of AWS. The model has demonstrated excellent discriminative ability, with AuROC of 0.82. Subsequently, an application was constructed to provide individualized recommendations for CT head scans based on each patient’s risk profile.

The number of CT head scans ordered by ED physicians has increased substantially [26]. Requesting unnecessary tests has been associated with overcrowding, patient severity, and low physician experience [27, 28]. Extensive research has consistently demonstrated a low yield of CT as a seizure work up tools and suggests a more conservative approach to the use of CT head scans based on clinical factors [5, 8, 29]. On the other hand, seizures represent clinically significant situations that require experienced assessment for accurate and timely patient guidance for further management. The triage strategies have been proposed to effectively reduced this type of problem [30]. Therefore, incorporating clinical prediction tools as part of the diagnostic triage process may enhance the management process, reduce unnecessary CT head scans, consequently leading to cost savings and reduced patient stay time in the ED [5, 8].

According to the result, the prevalence of observed positive finding on CT head scans was consistent with previous to the previous evidence [11]. Factors such GCS of 9–13 and ≤8, the presence of neurological deficits, and a history of malignancy have consistently demonstrated a strong association with positive lesions on CT head scans, as these factors exhibiting signs or increasing the risk of new brain lesions [5, 8]. Conversely, three predictors, including: a history of CVA, epilepsy, and the presence of alcohol withdrawal symptoms, decreased the risk of having positive findings. Patients with a history of CVA without new neurological deficits were likely to have seizures caused by old lesions [16]. Similarly, in the absence of either focal deficits on neurological examination or signs of acute head trauma, patients with alcohol withdrawal seizures or epileptic patient were likely to have normal findings on CT head scans [5, 31]. It is worth noting that CT head scans did not alter the acute management in these patients [8].

Our study represented the first attempt to construct a clinical decision support system for ordering CT head scans in non-traumatic patients presenting with seizures. The primary objective of this study was to balance between ensuring the standard care and the overuse of unnecessary CT head scans. We proposed a threshold set at a 20% probability. At this threshold, the model achieved a sensitivity of 77.5%, specificity of 79.5%, and a negative predictive value of 93.5%, indicating a well-balanced performance with the lowest false negative rate. With its excellent discriminative ability (AuROC of 0.82), the application offers a reliable tool for effectively reduce unnecessary use and instill confidence in supporting physicians in their decision-making process.

### Limitation

There are several important limitations that should be taken into account. Firstly, our model was developed using retrospective data. Missing data, or misclassification error made by the nature of retrospective data may introduce certain biases. Secondly, the assessment of some predictors such as focal neurological deficits and AWS was primarily conducted by general practitioners. The inherently subjective nature of these assessments raises concerns about potential inaccuracies. However, they represent real-world accuracies and baseline physician performance. Since our aim is to implement the prediction model primarily for use by general practitioners, particularly to assist less experienced physicians in decision-making. Additionally, the potential subjectivity in diagnosing AWS is relatively low, as most cases can be easily identified due to a clear history of heavy alcohol drinking followed by immediate discontinuation. Therefore, the future predictive performance is likely to be less impacted. Lastly, the models were constructed based on data from a single center. To guarantee the reliability of the model performance, it is advisable to conduct multicenter externally validated studies before integrating the diagnostic score into a wider clinical setting.

To access this application, we have provided the information as follows link: https://tharathipdevelop.com/pos-ct/form

## Supporting information

S1 File Supplementary S1 Table and S1, S2 Figs. (DOCX)

S2 File TRIPOD checklist. (PDF)

## References

[1] Tiamkao S, Sawanyawisuth K, Paowana W, Saengsuwan J, Arunpongpaisal S, Chaiyakum A, et al. Seizure presenting to the emergency department, Srinagarind Hospital. J Med Assoc Thai. 2006 Mar;89(3):362–7. . 16696421

[2] Martindale JL, Goldstein JN, Pallin DJ. Emergency department seizure epidemiology. *Emerg Med Clin North Am*. 2011;29:15–27 doi: 10.1016/j.emc.2010.08.002 21109099

[3] Pellock JM. Overview: Definitions and Classifications of Seizure Emergencies. *Journal of Child Neurology*. 2007;22(5_suppl):9S–13S. doi: 10.1177/0883073807303064 17690082

[4] ACEP Clinical Policies Committee; Clinical Policies Subcommittee on Seizures. Clinical policy: Critical issues in the evaluation and management of adult patients presenting to the emergency department with seizures. Ann Emerg Med. 2004 May;43(5):605–25. doi: 10.1016/S019606440400068X . 15111920

[5] Kvam KA, Douglas VC, Whetstone WD, Josephson SA, Betjemann JP. Yield of Emergent CT in Patients With Epilepsy Presenting With a Seizure. Neurohospitalist. 2019 Apr;9(2):71–78. doi: 10.1177/1941874418808676 Epub 2018 Nov 18. 30915184 PMC6429671

[6] Guerrini Renzo, and Barba Carmen, ’Classification, Clinical Symptoms, and Syndromes’, in Shorvon Simon, and others (eds), Oxford Textbook of Epilepsy and Epileptic Seizures, Oxford Textbook of (Oxford, 2012; online edn, Oxford Academic, 1 Dec. 2012), 10.1093/med/9780199659043.003.0007, accessed 3 Mar. 2024.

[7] Nowacki TA, Jirsch JD. Evaluation of the first seizure patient: Key points in the history and physical examination. Seizure. 2017 Jul;49:54–63. doi: 10.1016/j.seizure.2016.12.002 Epub 2016 Dec 8. . 28190753

[8] Burgess M, Mitchell R, Mitra B. Diagnostic testing in nontrauma patients presenting to the emergency department with recurrent seizures: A systematic review. *Acad Emerg Med*. 2022;29:649–657. doi: 10.1111/acem.14391 34534387

[9] Power SP, Moloney F, Twomey M, James K, O’Connor OJ, Maher MM. Computed tomography and patient risk: facts, perceptions and uncertainties. World J Radiol. 2016;8:902–915. doi: 10.4329/wjr.v8.i12.902 28070242 PMC5183924

[10] Griffey RT, Jeffe DB, Bailey T. Emergency physicians’ attitudes and preferences regarding computed tomography, radiation exposure, and imaging decision support. Acad Emerg Med. 2014 Jul;21(7):768–77. doi: 10.1111/acem.12410 . 25125272 PMC4135442

[11] Reinus William R, Wippold Franz J, Erickson Kavita K, Seizure patient selection for emergency computed tomography, Annals of Emergency Medicine, Volume 22, Issue 8, 1993, Pages 1298–1303, ISSN 0196-0644, doi: 10.1016/s0196-0644(05)80111-8 8333632

[12] Hui AC, Tang A, Wong KS, Mok V, Kay R. Recurrence after a first untreated seizure in the Hong Kong Chinese population. Epilepsia 2001;42:94–97 doi: 10.1046/j.1528-1157.2001.99352.x 11207791

[13] Mabaso SH, Bhana-Nathoo D, Lucas S. An audit of CT brain findings in adults with new-onset seizures in a resource restricted setting in South Africa. SA J Radiol. 2022 Jan 20;26(1):2294. doi: 10.4102/sajr.v26i1.2294 . 35169503 PMC8831926

[14] Collins GS, Reitsma JB, Altman DG, Moons KG. Transparent reporting of a multivariable prediction model for individual prognosis or diagnosis (TRIPOD): the TRIPOD statement. BMJ. 2015 Jan 7;350:g7594. doi: 10.1136/bmj.g7594 . 25569120

[15] Huff JS, Murr N. Seizure. [Updated 2023 Feb 7]. In: StatPearls [Internet]. Treasure Island (FL): StatPearls Publishing; 2023 Jan-. https://www.ncbi.nlm.nih.gov/books/NBK430765/

[16] Myint PK, Staufenberg EFA, Sabanathan K. Post-stroke seizure and post-stroke epilepsy. Postgrad Med J. 2006;82(971):568–72. doi: 10.1136/pgmj.2005.041426 16954451 PMC2585721

[17] Wippold FJ 2nd; Expert Panel on Neurologic Imaging. Focal neurologic deficit. AJNR Am J Neuroradiol. 2008 Nov;29(10):1998–2000. . 19008324 PMC8118959

[18] Thijs RD, Surges R, O’Brien TJ, Sander JW. Epilepsy in adults. Lancet. 2019 Feb 16;393(10172):689–701. doi: 10.1016/S0140-6736(18)32596-0 Epub 2019 Jan 24. . 30686584

[19] Diagnostic and Statistical Manual of Mental Disorders. 5th ed. Washington, DC: American Psychiatric Association; 2013.

[20] Kattimani S, Bharadwaj B. Clinical management of alcohol withdrawal: A systematic review. Ind Psychiatry J. 2013 Jul;22(2):100–8. doi: 10.4103/0972-6748.132914 . 25013309 PMC4085800

[21] Riley RD, Snell KI, Ensor J, Burke DL, Harrell FE Jr, Moons KG, et al. Minimum sample size for developing a multivariable prediction model: PART II—binary and time-to-event outcomes. Stat Med. 2019 Mar 30;38(7):1276–1296. doi: 10.1002/sim.7992 Epub 2018 Oct 24. Erratum in: Stat Med. 2019 Dec 30;38(30):5672. . 30357870 PMC6519266

[22] Chowdhury MZI, Turin TC. Variable selection strategies and its importance in clinical prediction modelling. Fam Med Community Health. 2020;8(1):e000262. doi: 10.1136/fmch-2019-000262 32148735 PMC7032893

[23] Hosmer DW, Lemeshow S (2000). Applied logistic regression, 2nd ed. Wiley, pp 156–164

[24] Vickers A.J.; Elkin E.B. Decision Curve Analysis: A Novel Method for Evaluating Prediction Models. Med. Decis. Mak. Int. J. Soc. Med. Decis. Mak.2006,26, 565–574. doi: 10.1177/0272989X06295361 17099194 PMC2577036

[25] Vickers A.J., van Calster B. & Steyerberg E.W. A simple, step-by-step guide to interpreting decision curve analysis. Diagn Progn Res 3, 18 (2019). doi: 10.1186/s41512-019-0064-7 31592444 PMC6777022

[26] Hess EP, Haas LR, Shah ND, Stroebel RJ, Denham CR, Swensen SJ. Trends in computed tomography utilization rates: a longitudinal practice-based study. J Patient Saf. 2014 Mar;10(1):52–8. doi: 10.1097/PTS.0b013e3182948b1a . 24080717

[27] Koch C, Roberts K, Petruccelli C, Morgan DJ. The Frequency of Unnecessary Testing in Hospitalized Patients. Am J Med. 2018 May;131(5):500–503. doi: 10.1016/j.amjmed.2017.11.025 Epub 2017 Dec 7. . 29224739 PMC8628817

[28] Miyakis S, Karamanof G, Liontos M, Mountokalakis TD. Factors contributing to inappropriate ordering of tests in an academic medical department and the effect of an educational feedback strategy. Postgrad Med J. 2006 Dec;82(974):823–9. doi: 10.1136/pgmj.2006.049551 . 17148707 PMC2653931

[29] Salinsky M, Wong VSS, Motika P, Meuse J, Nguyen J. Emergency department neuroimaging for epileptic seizures. Epilepsia. 2018;59:1676–1683. doi: 10.1111/epi.14518 30019464

[30] Yarmohammadian MH, Rezaei F, Haghshenas A, Tavakoli N. Overcrowding in emergency departments: A review of strategies to decrease future challenges. J Res Med Sci. 2017 Feb 16;22:23. doi: 10.4103/1735-1995.200277 . 28413420 PMC5377968

[31] Feussner JR, Linfors EW, Blessing CL, Starmer CF. Computed tomography brain scanning in alcohol withdrawal seizures. Value of the neurologic examination. Ann Intern Med. 1981 Apr;94(4 pt 1):519–22. doi: 10.7326/0003-4819-94-4-519 . 7212510

