# Supplementary material for: Development of clinical prediction model to guide the use of CT head scans for non-traumatic Thai patient with seizure: A cross-sectional study
Source: PLoS One. 2024 Jul 10;19(7):e0305484. doi: 10.1371/journal.pone.0305484 (PMC11236092; doi:10.1371/journal.pone.0305484)
Supplement: S1 File — (DOCX) [file pone.0305484.s001.docx]

**Figure 1S.** Calibration plot

**
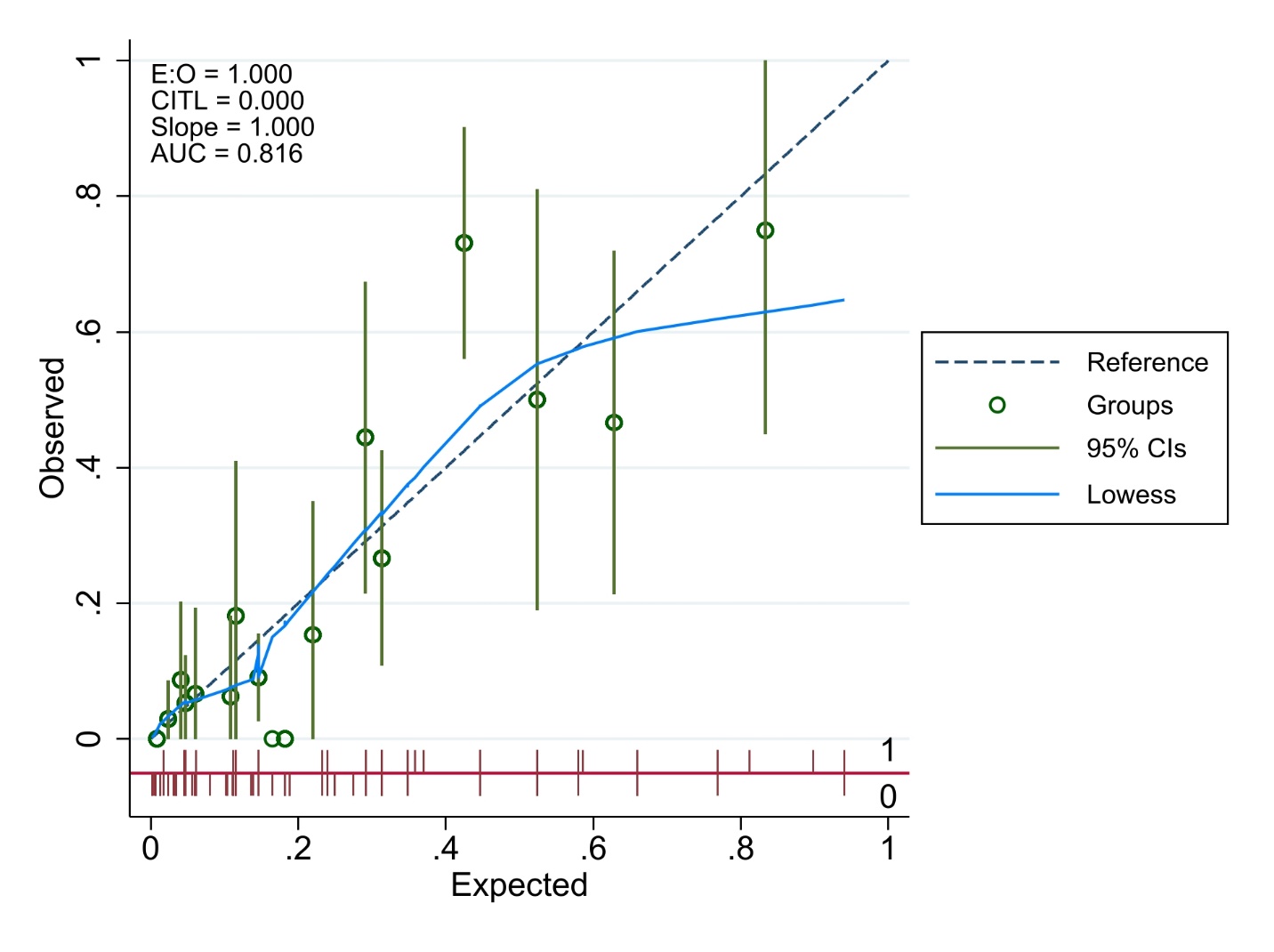
**

**Figure 2S.** Bootstrap calibration plot**,** E:O ratio, calibration Slope, and C-statistic

**
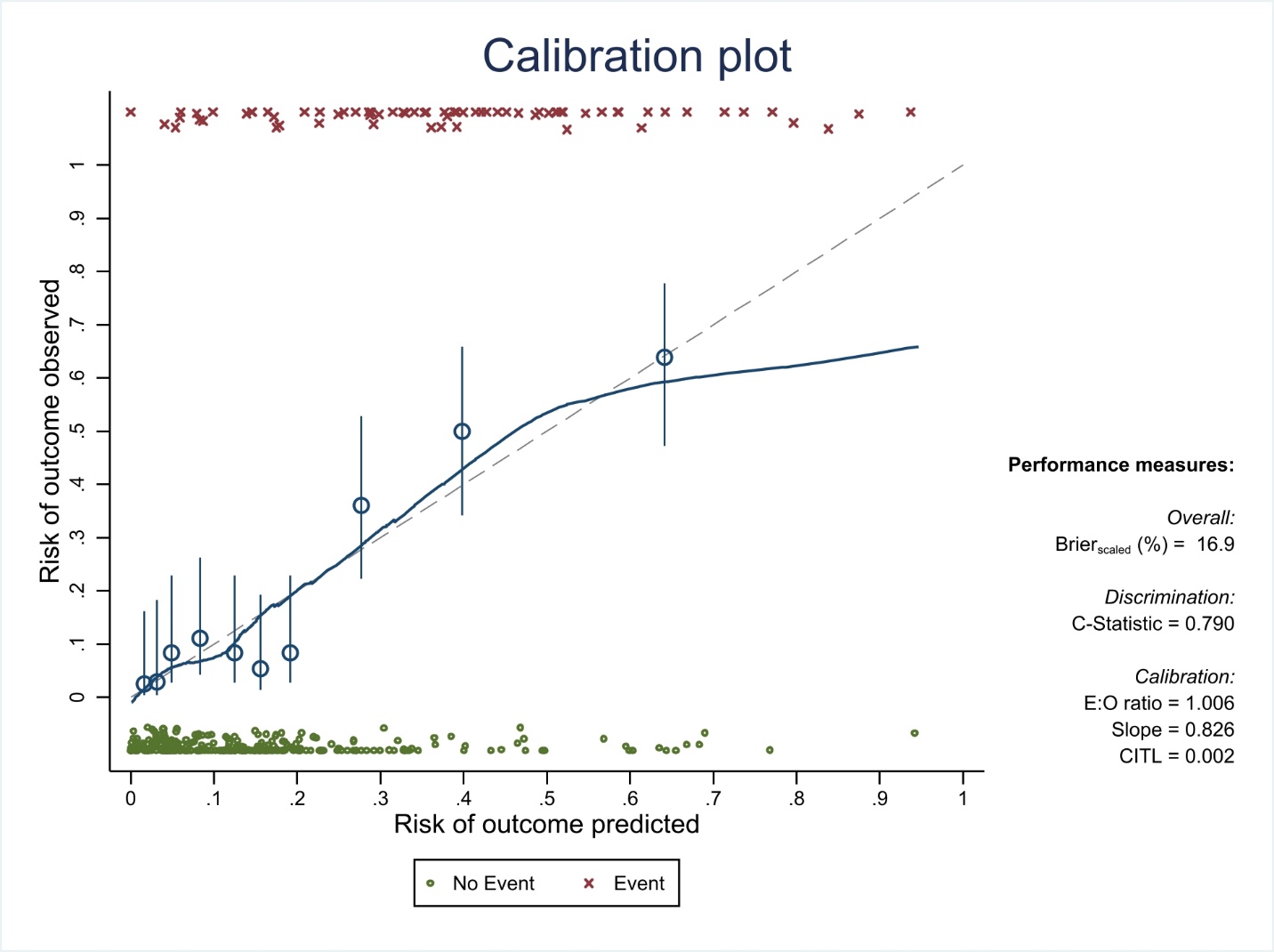
Table 1S:** Univariable logistic regression analysis for prediction of positive finding on CT head

| **Parameters** | **Crude OR** | **95% CI** | **p-value** |
| --- | --- | --- | --- |
| Male | 0.63 | 0.36 - 1.11 | 0.108 |
| Age | 1.01 | 1.00 - 1.03 | 0.092 |
| Altered mental status |  |  |  |
| - GCS >13 | reference | - |  |
| - GCS 9-13 | 2.09 | 1.10 - 3.95 | 0.024 |
| - GCS ≤8 | 4.18 | 2.18 - 8.02 | <0.001 |
| Focal neurological deficit | 4.49 | 2.47 - 8.14 | <0.001 |
| Previous CT status |  |  |  |
| - Normal previous CT | reference | - |  |
| - No previous CT | 2.53 | 1.20 - 5.34 | 0.014 |
| - Abnormal finding | 2.05 | 0.90 - 4.66 | 0.086 |
| History of malignancy | 9.34 | 3.10 - 28.41 | <0.001 |
| History of CVA | 0.43 | 0.20 - 0.95 | 0.037 |
| Epilepsy | 0.24 | 0.10 - 0.58 | 0.002 |
| Alcoholic withdrawal symptoms | 0.13 | 0.03 - 0.52 | 0.004 |

Abbreviation: crude OR, unadjusted odds ratio; CVA, cerebrovascular accident; GCS, Glasgow coma scale; 95%CI, 95% confident interval
